# Co-design and Development of Implementation Strategies: Enhancing the PAX Good Behaviour Game in Australian Schools

**DOI:** 10.1007/s10935-023-00749-9

**Published:** 2023-09-23

**Authors:** Rachel Baffsky, Rebecca Ivers, Patricia Cullen, Lauren McGillivray, Aliza Werner-Seidler, Alison L. Calear, Philip J. Batterham, John W. Toumbourou, Rhoni Stokes, Pauline Kotselas, Traci Prendergast, Michelle Torok

**Affiliations:** 1 School of Population Health, University of New South Wales, Sydney, Samuels Building, F25, Samuel Terry Ave, Kensington, NSW Australia; 2 Black Dog Institute, University of New South Wales, Sydney, Australia; 3 Centre for Mental Health Research, The Australian National University, Acton, ACT Australia; 4 School of Psychology and Centre for Social and Early Emotional Development (SEED), Deakin University, Geelong, VIC Australia; 5 Department of Education, New South Wales (NSW), Parramatta, NSW Australia

**Keywords:** Co-design, Teachers, School-based programs, Implementation science, Mental health

## Abstract

Effective implementation strategies are important for take-up of programs in schools. However, to date, few implementation strategies have been co-designed with teachers and support staff (including principals) in Australia. The aim of this study was to iteratively co-design multiple implementation strategies to enhance the delivery of mental health prevention program, PAX Good Behaviour Game, in New South Wales primary schools. The secondary aim was to evaluate the acceptability of the implementation strategies from the perspective of school staff. Twenty-nine educational staff (including principals) informed the co-design of the implementation strategies across three phases. Phase 1 involved a rapid review of the literature and stakeholder meetings to agree upon potential evidence-based strategies. Phase 2 involved focus group discussions with educational staff to co-design implementation strategies. Phase 3 involved semi-structured interviews with school staff to assess strategy acceptability after implementation at 6-months post-baseline. Data were analysed using deductive, framework analysis. The final co-designed intervention included nine implementation strategies accessible through a toolkit delivered to the school’s leadership team. These strategies were deemed acceptable in school settings that experienced periods of both face-to-face and remote learning due to the changing COVID-19 situation in 2021. This paper contributes to the implementation literature by transparently reporting how educational staff-informed implementation strategies were iteratively co-designed. This will provide a roadmap for other researchers to co-design implementation strategies to further support the delivery of evidence-based prevention programs in schools.

**Supplementary Information:**

The online version contains supplementary material available at 10.1007/s10935-023-00749-9.

## Background

It is estimated that half of all adult mental health conditions emerge by the age of 14 (Kessler et al., 2007), and so schools are considered key settings for mental health prevention. While the risk of children and adolescents developing these conditions can be reduced via school-based early prevention programs (Werner-Seidler et al., 2021), there is increasing evidence that educators experience many barriers to implementation which undermine potential benefits (Williams et al., 2020). While not exhaustive, these barriers include time constraints (Allara et al., 2019), lack of training, high staff turnover (Dijkman et al., 2017), misalignment between program and regular practice (Coombes et al., 2016), program implementation fatigue, prioritising academic learning over socio-emotional learning (Locke et al., 2019), lack of principal support, lack of financial and/or material resources (Herlitz et al., 2020) and educational policy constraints (Owens et al., 2014). Furthermore, school staff typically do not receive expert support or consultation on how to collect and analyse data to guide and improve program implementation (Bruhn et al., 2015).

To support prevention programs being embedded into schools, there is a need for implementation strategies to be developed and tested for efficacy (Lyon & Bruns, 2019). Implementation strategies are activities, processes, or resources that can be tailored to improve the implementation outcomes of evidence-based programs, which in turn enhance mental health outcomes (Proctor et al., 2009). There are eight implementation outcomes that predict mental health benefits: reach, adoption, sustainability, fidelity, cost, appropriateness, acceptability, and feasibility (Proctor et al., 2011). Implementation strategies can manipulate four types of implementation factors: system/environment (educational policy), organisational (whole-school setting), groups/teams of staff, and individual program providers (teachers) (Proctor et al., 2009).

In educational settings, there is emerging evidence to suggest that monitoring program delivery and providing feedback to staff directly involved in implementation (Solomon et al., 2012), distributing educational materials that explain the program (Nathan et al., 2016), recognition of effort (Wolfenden et al., 2019), executive leadership support (Baffsky et al., 2022b) and school champions (Dijkman et al., 2017; Nadeem et al., 2018) enhance implementation when used in combination with other strategies such as providing ongoing training (Sutherland et al., 2020) and coaching (Smith et al., 2018). To date, most school-based implementation studies have focused on physical health programs, and the majority have been conducted in the United States (US) (Barnes et al., 2021).

Strategies that work in other countries (e.g., US) may not work in Australia, due to unique operational and strategic differences between schools. First, the Australian curriculum is considered relatively crowded (Bowles et al., 2017) and mandates teaching socio-emotional skills (Australian Curriculum, 2018). However, there is no guidance on how these skills can be integrated into subject learning to protect teachers’ work hours (Gilbert, 2019), which are already higher than teachers in other OECD countries (Thomson & Hillman, 2019). Second, Australia’s national educational policy lacks targets for socio-emotional learning, such that it is often under prioritised (Productivity Commission, 2020). Third, Australia does not have national or state-level policy guiding standardised mental health program implementation, as seen in the US (Laurens et al., 2021). Instead, the onus is on Australian principals to select evidence-based programs and develop their own implementation supports (Collie et al., 2017), adding to their already high administrative workload (Thomson & Hillman, 2019). To overcome these implementation challenges, the Australian Productivity Commission has recommended schools consider teacher training, coaching, monitoring program progress, and the use of dedicated program champions to support the uptake of evidence-based mental health programs (Productivity Commission, 2020).

To address implementation knowledge gaps, we are conducting a ‘hybrid type 3’ effectiveness-implementation trial (Curran et al., 2012) of an evidence-based social-emotional universal prevention program, PAX Good Behaviour (PAX GBG), in New South Wales (NSW) government primary schools, Australia. The PAX GBG is a classroom-based intervention, designed to support the development of emotion and behavioural regulation, and teach prosocial decision-making (Simpson et al., 2020). The program consists of 10 evidence-based and trauma-informed strategies that teachers introduce into regular lessons. The first strategy sets expectations for appropriate classroom behaviour (called PAX behaviours) and inappropriate behaviours (called ‘spleems’). The other strategies encourage PAX behaviours and discourage spleems. Once students master the individual strategies, they are combined and simultaneously delivered as a ‘PAX Game’. During the game, students work in teams on an academic task for a fixed time. Teams who display four or less spleems at the end of the time period are rewarded collectively.

The standard PAX GBG model is supported by teacher training, coaching, a classroom toolkit to deliver strategies and a printed instructional manual. The 6–8 h teacher training is delivered by an expert from PAXIS, the US-based program developers, before implementation and entails strategy information and modelling. The physical program resources and instruction manual are distributed. Coaching is provided by the PAXIS program experts as needed during the first year of implementation and involves consultations to resolve local challenges. The PAX GBG and its standard implementation process are outlined in a logic model in Coombes et al. (2016).

Our PAX GBG trial is described in detail in the study protocol (Baffsky et al., 2022a). Briefly, we are using a cluster randomised controlled design to test if an evidence-informed multicomponent implementation strategy (called ‘PAX Plus’) leads to higher rates of program adoption compared to the standard delivery model. PAX Plus is a toolkit offering access to nine evidence- and user-informed implementation strategies in addition to the training and educational materials provided in the standard model. Schools were randomly allocated PAX Plus (intervention) or standard delivery (control) upon trial registration. The current study reports on the co-design of the PAX Plus strategy with educational staff (end users).

There have been some efforts, mainly in the US, to co-design strategies to enhance the implementation of evidence-based mental health prevention programs. For example, the standardised PAX GBG coaching was iteratively developed with input from teachers and students (Becker et al., 2013). Other school-based mental health programs (Bruns et al., 2016; DuPaul et al., 2018), and their implementation supports such as educational materials (Coelho et al., 2016), training (Kern et al., 2015), and implementation manuals (Coles et al., 2015) have been informed by feedback from teachers, parents and/or school mental health professionals.

Outside of the US, researchers have worked with health departments to select and adapt PAX GBG and its implementation supports to be culturally appropriate for different countries. In Brazil (Schneider et al., 2016), Estonia (Streimann et al., 2017), England (Coombes et al., 2016) and the Netherlands (Breeman et al., 2016) the language of PAX GBG was adapted to fit local context. An extended 3-day training was used in Estonia (Streimann et al., 2017) and the widely available PAXIS 1-day refresher training utilised in England (Coombes et al., 2016). In Canada, training was modified to include relatable examples of delivering PAX GBG in First Nations communities (Wu et al., 2019). In Sudan, educators and parents worked to co-design rewards they perceived would be more acceptable to students than the standard US rewards (Saigh & Umar, 1983). In Chile, regular team meetings were used to facilitate cultural adaptations of the PAX GBG during early adoption in schools (Pérez et al., 2005).

Whilst it is common for researchers to acknowledge that stakeholder feedback guided intervention development or refinement, studies rarely described co-design processes in sufficient detail to allow quality assessment, learning, or replication. Furthermore, researchers frequently report that the co-design process improved a program’s acceptability, feasibility, adoption, effectiveness, sustainability (Kern et al., 2015) or fit to context (Becker et al., 2013; Bruns et al., 2016) without substantiating these results with data.

To address these gaps, this study reports the process in which a multicomponent implementation strategy (PAX Plus) was co-designed with educational staff in 15 NSW government primary schools prior to PAX GBG implementation, and then refined following acceptability testing 6-months into the trial.

## Methods

### Study Design and Ethics

This study used a transdisciplinary action research approach, in which researchers and educators shared power in co-developing the PAX Plus implementation strategy (Stokols, 2006). The development process was guided by Hawkins et al.’s (2017) framework for the co-design of public health interventions, using a qualitative methodology (Hawkins et al., 2017). Ethics approval for this study was granted by the University of New South Wales Human Research Ethics Committee (HC200759) and the NSW Government State Education Research Applications Process (SERAP 2020364).

### Participants and Setting

The 15 New South Wales government primary schools involved in the co-design were registered in the wider trial, with plans to have staff trained in PAX GBG. Twelve schools were regional (80%) and three were urban (20%). School sizes ranged from 20–437 students (see supplementary file 1 for further details). From these 15 schools, we worked with 29 educational staff, 13 of whom were teachers, 16 were part of the school leadership team, and 27 were female. Their roles and involvement in the co-design process are described in Supplementary File 1.

### Co-design Procedure

**Fig. 1:**
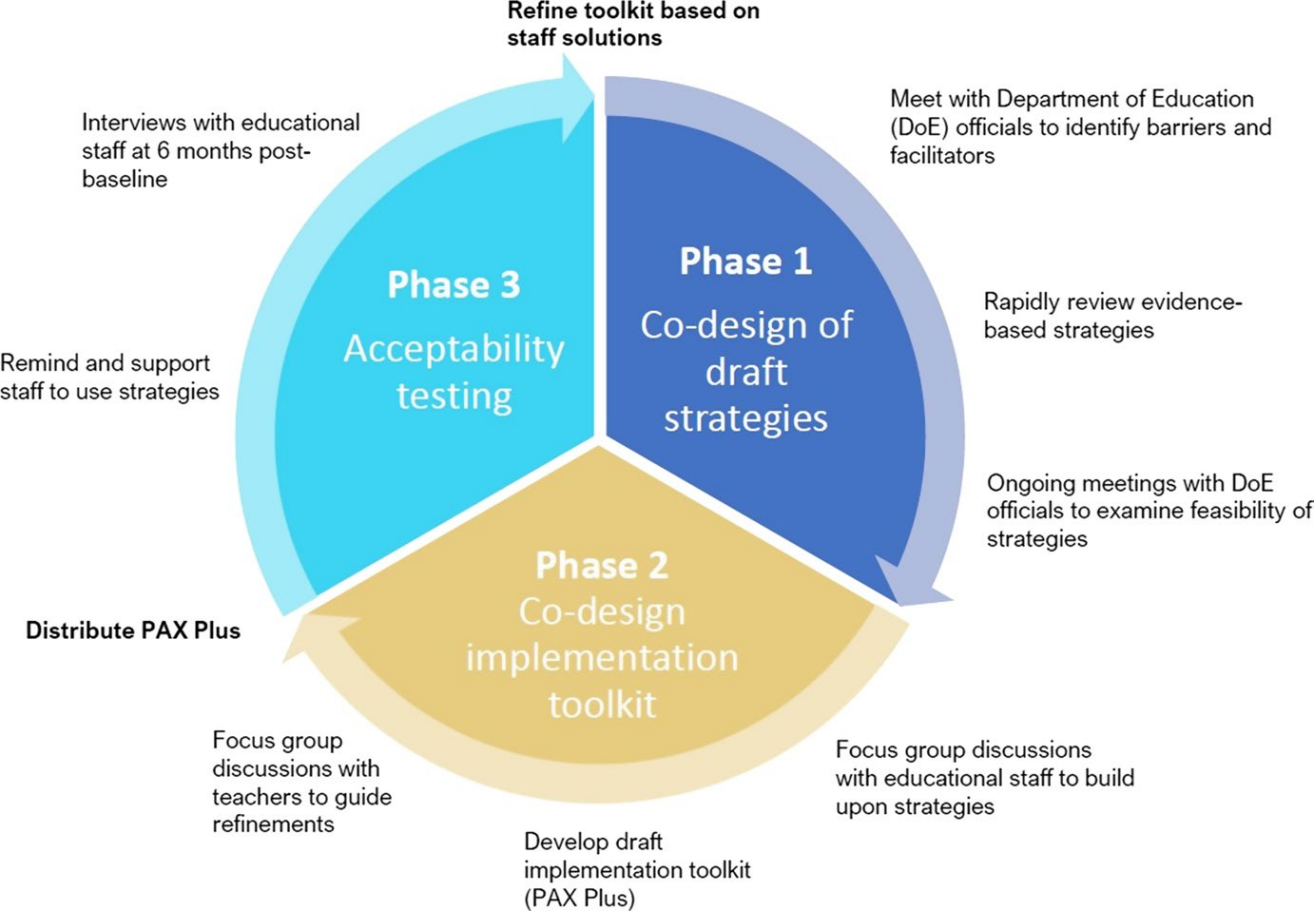
Three co-design phases and overview of methods

Figure 1 shows the methods used across the three co-design phases in our study.

#### Stage 1: Co-design of draft strategies

Two researchers (RB and MT) worked with the NSW Department of Education wellbeing leadership team (co-authors RS, PK, TP) at fortnightly (every two weeks) meetings for 3 months to shortlist potential strategies for inclusion in the toolkit. The Department of Education is responsible for funding and distributing resources to support the program. Meetings focused on discussing the feasibility of strategies with the strongest evidence of improving program implementation in schools. Strategies were identified through a rapid review of the academic and grey literature (original studies, review studies) published from 1 January 2000-28 January 2021. Four electronic databases (PubMed, PsycINFO, CINAHL and ERIC) and Google Scholar were searched. The inclusion criteria were: (a) Population—educators (teachers and school leadership staff), (b) Intervention—a strategy to support implementation of an evidence-based mental health prevention program for children in primary schools, (c) Study designs—randomised controlled trials, quasi-experimental studies, pretest-post-test studies and qualitative studies, (d) Implementation outcome(s)—adoption, reach, fidelity, sustainability, appropriateness, acceptability or feasibility, (e) Publication type—peer-reviewed journal articles, (d) Language—English. RB single-screened titles and abstracts and full texts against these inclusion criteria in a Microsoft excel spreadsheet. See supplementary file 2 for the full search strategy.

#### Stage 2: Co-design of the implementation toolkit

Three focus group discussions (FGDs) with educational staff were conducted to discuss and build upon the strategies drafted in Stage 1. A NSW Department of Education partner (RS) used purposive sampling to identify staff who had adopted PAX GBG and invited them to attend the FGDs. Informed ‘opt-in’ consent was obtained via email. RB and RS co-facilitated the FGDs, using a semi-structured interview guide. The guide provided a brief overview of the six strategies identified in phase 1 and their evidence-base followed by questions about acceptability, feasibility, and optimal delivery model. A sample question about the ideal delivery mode for audit and provide feedback was, “What type of feedback would you find most motivating (if any)?”. Lastly, the guide included open-ended questions about additional strategies educational staff wanted to use to overcome implementation challenges, to capture new ideas. For example, “What other strategies (not discussed) could help you to implement the PAX GBG?”.

There were six staff in the first focus group, five in the second and five in the third (supplementary file 1). Each focus group lasted 45–60 min and took place between 25 February 2021 and 9 March 2021. FGDs were conducted online using the Microsoft Teams videoconferencing platform.

Findings from FGDs informed the development of the first iteration of the PAX Plus implementation toolkit, which consisted of eight implementation strategies (see Table 1). The toolkit described the rationale and evidence (if applicable) for each included strategy, and the actions required by schools to support its implementation.

**Table 1:** The eight strategies comprising the ‘PAX Plus implementation toolkit’ with descriptions and exemplar quotes from FGD (*N* = 16)

Strategy	Description	Quote
Remind school personnel	The researchers developed fortnightly e-newsletters containing practical tips and reminders for delivering PAX GBG program components	*“The newsletters and all of that are fabulous for a resource…And the website itself has got an amazing amount of resources there that you can use, but again…people are time poor…there are people that just want to be able to short circuit…these are the must dos, these are the can do’s.*” P12, Principal, regional school
Peer learning network	The NSW Department of Education set up a Teams Channel for educational staff to learn from other educators delivering PAX GBG in NSW schools	*“I was part of [deidentified wellbeing program] for a long time, and I really took a lot from the network meetings, because we would hear strategies of other schools. So, I know we have so many network meetings but truly somewhere where we could meet other teachers that are also doing PAX and we could share our success stories, or we could troubleshoot that would be amazing.”* P6, Assistant Principal/teacher, regional school
Promoting PAX Chats	The NSW Department of Education email teachers reminders to participate in PAX Chats, which are quarterly online consultations delivered by US program developers (PAXIS)	*“I think it's a great benefit, because then you can look and see what other people are doing and what they need help with.”* P14, Principal, regional school *“As far as the PAX chats it’s just time poor, no having the time set aside or remembering to participate in them.”* P11, Teacher, regional school
Continuous progress monitoring	Principals encouraged to provide teachers with a fidelity self-checklist and administer a monthly online self-report survey to assess the frequency and fidelity of program implementation	*“I think that a checklist would be quite handy and then I guess that collection of data from an external person, other than the school has an unbiased view, then, to say you know 80% of the teachers across who have had PAX training are implementing you know X, Y and Z strategies.”* P14, Principal, regional school
Executive leadership support	The leadership team receive a 15-min monthly support call from the researcher	[regarding executive support] “*I can't stress how important it is from that top down support at the principal level to then go this is a benefit to you, to your teaching staff to your students or whatever, it's that the bottom line is it's going to make a difference in the classroom, teachers, and it's going to make a difference for your kids.”* P11, Principal, regional school
Program champions	A self-selecting program advocate who provides other staff with information and motivation to support implementation	*“If you had a criteria that you could give to schools to say these are the things that you would be needing to do as a representative that would be helpful for us to hand out to staff to say is anybody interested in doing that and I guess if there was no one…then it would then fall back to either one of us on the leadership team.”* P14, Principal, regional school
Recognition system	The principal is encouraged to use ‘tootle boards for teachers’ and/or certificates of achievement to publicly recognise educators for their PAX GBG support	*“We put a tootle board up in our staff room. So, every single week we have it's a blank board that just says tootles. You would be amazed that every single week the board is covered in affirmations… so staff can begin to recognise and be thankful for the things that other staff members do.”* P11, Principal, regional school *“As part of our [deidentified wellbeing program] we currently have a staff appreciation block and each week, teachers are given a little certificate… we find that motivating and it’s something that we already do but we’re probably going to incorporate the PAX side of things.”* P4, Teacher, urban school
Audit and provide feedback	Principals are encouraged to monitor the effect of PAX GBG on students’ outcomes and feed this information back to teachers as a motivational tool. Student outcomes can be measured using aggregate school data such as attendance rates or behavioural incidents or the PAX Scoreboard which tracks the number of inappropriate behaviours during a PAX Game	*“I think the data speaks for itself, like that's what we strive towards…as a teacher, I just want to see my kids thriving so I’m gonna see those benefits in my classroom that's what I need.”* P6, Teacher, regional school In response to question of how we can monitor the success of the program: *“Having that extra support with the analysis of the data that's already within the school so schools would have data already for maybe central incidents classroom incidents.”* P5, Teacher, urban school

#### Stage 3: Acceptability testing (prototyping)

Following the roll out of PAX GBG in schools and the intervention group receiving PAX Plus implementation toolkit for six months, RB conducted interviews with educational staff to identify early issues with the acceptability of PAX Plus, and to guide refinements. The interviews took place between 25 November and 16 December 2021. Using purposive sampling, RB identified staff from different roles (teacher, principal, assistant principal, instructional leader) and varying geographical locations. Instructional leaders are staff who mentor and train teachers to support student learning. All staff consented to participate and record interviews. Interviews were directed by a semi-structured interview guide asking staff to identify strategies that worked to support PAX GBG in the past 6 months. Staff in the intervention group were asked about the acceptability and feasibility of the initial PAX Plus strategies, and to recommend changes. A sample interview question was, “To what extent did you find the e-newsletters appealing (if at all)?” Staff were asked to suggest new strategies, not included in the original toolkit that may be important to program implementation. Suggested strategies were tested for acceptability/feasibility in subsequent interviews. Each interview lasted between 35 and 50 min, and staff were reimbursed with a $40 e-gift voucher. The staff-informed refinements were formalised in version 2.0 of the PAX Plus implementation toolkit in January 2022, and then trialled in a second cohort of schools.

### Data Analysis

Focus group discussions and interviews were analysed using a deductive framework analysis approach (Gale et al., 2013). Transcripts were managed and stored on NVIVO software (QSR International, 2020). RB performed line-by-line deductive coding of the phase 2 FDGs, which involved generating codes that related to strategies identified in the rapid review (phase 1) or identified through this data. These codes were clustered into two categories a priori: (i) School-level strategies and (ii) Teacher-level strategies, and then defined in an analytic framework. The analytic framework was used to deductively code the phase 3 interview data and was also iteratively refined based on interview data about what strategies worked in practice. The analytic framework was reviewed and refined in discussions between RB, MT, and PC resulting in nine codes/strategies.

## Results

**Fig. 2:**
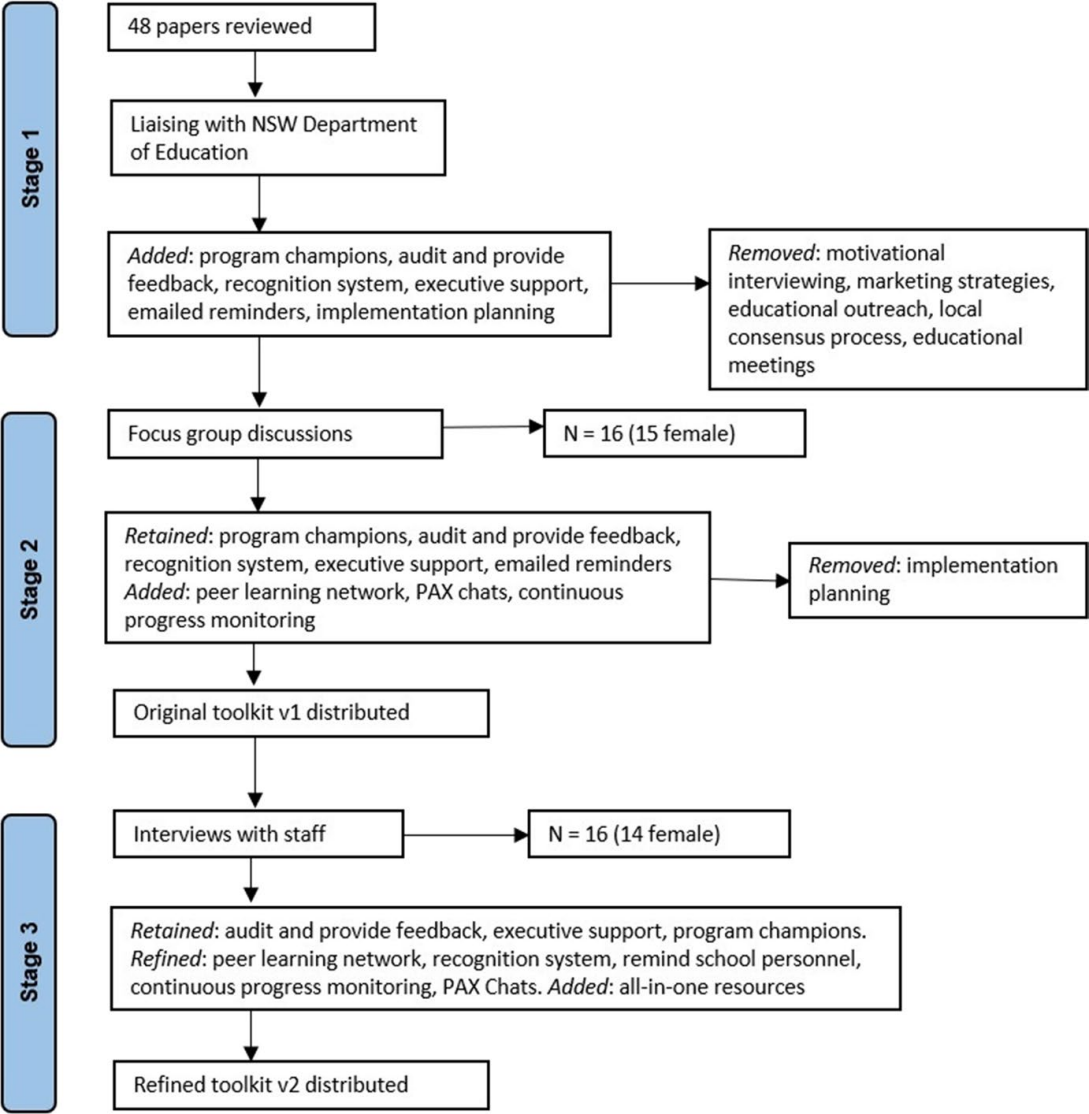
Overview of the findings from the three stages of the co-design process

Figure 2 presents an overview of the strategies from the staged co-design process. These will be described below.

### Stage 1: Co-design of draft strategies

The rapid review screening process is outlined in supplementary file 3. Forty-eight eligible papers were included, and eleven effective strategies identified (Supplementary file 4). In collaboration with the Department of Education, we shortlisted six strategies for potential inclusion in the toolkit: using a program champion, audit and provide feedback, recognition system, executive support, emailed reminders, and implementation planning. The most common reasons for exclusion of strategies were that they were too resource intensive in respect to personnel (e.g., coaching, consultation) and/or funding.

### Stage 2: Co-design of the implementation toolkit

Sixteen educational staff agreed upon eight strategies in focus groups, which formed version 1.0 of the PAX Plus implementation toolkit, sent to intervention schools of the wider trial in July 2021. Staff contributions to the selection, format and delivery mode of strategies are described below (and summarised in Table 1).

1. Strategies

Staff wanted five out of the six strategies from Phase 1 to be included in the toolkit. They did not perceive implementation planning to be useful but agreed it could be integrated into ‘executive support’. Three additional strategies were put forward in the FGDs. First, a peer learning network to exchange knowledge with staff from other NSW schools. Second, access to PAX Chats, which were ongoing online consultation sessions about specific program components administered by PAXIS. Third, a continuous progress monitoring system for quality improvement.

(2) Format of delivery

Formatting recommendations were based on educators’ reports of what had worked to support other programs in practice. They recommended using ‘tootle boards for teachers’ and/or ‘certificates of achievement’ as recognition systems (Table 1). The ‘tootle board for teachers’ is a blank board in the staff room to post affirmations (tootles notes) acknowledging peers’ PAX GBG implementation efforts. It was suggested that schools use existing administrative data, such as behavioural incident referrals, as evidence of the impact of PAX GBG on students and provide this feedback to teachers as a motivational tool. Some staff also felt it would be useful to have ‘fidelity’ self-report checklists and brief ‘process’ surveys for continuous progress monitoring. It was raised that the ‘school champion’ should be self-selecting (rather than chosen by the school leadership) and ideally positioned within the leadership team.

(3) Timing

Educational staff explained they had limited time outside of class to support PAX GBG and recommended implementation activities be paced more slowly than suggested in Phase 1. They recommended executive support meetings and e-newsletters (emailed reminders) be provided monthly, compared to fortnightly as suggested by the Department of Education. They also suggested program champions organise fortnightly implementation team meetings with school personnel, rather than weekly meetings as suggested by the researchers.

### Stage 3: Acceptability Testing (prototyping)

Nineteen educational staff agreed to be interviewed at the 6-month follow up, however, three withdrew prior to the interview due to time constraints. Table 2 summarises the acceptability of the original PAX Plus implementation toolkit to staff, based on majority opinion. It was consistently reported that program champions, audit and provide feedback, and executive support were acceptable and could continue to be implemented as originally designed. Educational staff liked some components of the training and recognition system strategies, such as learning from experts and positive reinforcement, however thought that the format and timing could be improved. The PAX Chats, peer learning networks, e-newsletters and continuous progress monitoring strategies were underutilised by all educational staff, with potential to be improved through reformatting or strategy promotion.

**Table 2:** Educational staff feedback (*N* = 16) on the acceptability of original toolkit

Strategy	Feedback	Description	Illustrative quote
Program champions	Acceptable	Participants liked having program champions to support teachers’ capacity building through role modelling and providing feedback	[in response to question what would work to improve implementation] *“Just timetabling the leader that we want to lead PAX in the school, providing her an hour a week to lead the initiative and hopefully that person going into classrooms, demonstrating it, providing feedback, supporting teachers with professional development and being the role model for the school.”* P23, Instructional Leader, regional school
Audit and provide feedback	Acceptable	Principals appreciated the value of using existing evaluation tools to track changes in student wellbeing during PAX program implementation	*“We have been using another tool called Pulse Educator…. It's a weekly check-in *via* an app for all the students [Years] 3–6… the analytics from that would be great to be able to crosscheck with the classes where you know they've been really implementing PAX to see what the students are saying.”* P10, Principal, regional school
Executive support	Acceptable	Principals and school champions felt the monthly meetings with the researcher triggered a sense of accountability to stay on track with the planned roll out of PAX GBG. They also found it useful to have the researcher’s outsider perspective in problem-solving implementation challenges	*“The monthly meetings are good to keep you focused and keep us honest and make sure that it is happening. So, probably it's been good to keep us on track…Just keeping it alive to us and keeping everything moving.”* P27, Teacher, regional school *“It's having another person [the researcher] looking and just gives you a different perspective, I think. We've used a lot of the things that you've suggested…just having that extra guidance was good for us because it's like, oh, this is how we can get back on track.”* P28, Principal, regional school
Recognition system	Partially acceptable	Few schools used the recommended recognition systems (certificates of achievement/tootle boards). Staff that did receive recognition felt this positive reinforcement motivated them to continue to implement the program	*“[a recipient of a certificate] He felt appreciated…And he just was like, oh, thank you very much. So, it's giving credence to people that are doing things for you that they don't have to, and that's a nice thank you.”* P28, Principal, regional school *“[the tootle board for teachers] It actually makes you aware of what's going on and who's helping who…. It's really nice for pointing out all the positives and all the good things that are happening that people are doing…Yes, it definitely is [motivating]. It's a really nice feeling when you go in and see that someone's pointed out something that you've done and either thanked you for it or just written it up there so everybody else can see. It is really nice.”* P22, Assistant Principal, regional school
PAX Chats	Underutilised	PAX Chats were underutilised. Time and the competing demands of the academic curriculum were consistently identified as a barrier to teachers’ participating in PAX Chats	When asked why they had not attended PAX Chats one teacher explained: *“I mean it’s been a time thing. Everyone’s just been really, really flat-out with getting lessons out.”* P27, Teacher, regional school
Peer learning network	Underutilised	Staff consistently asked for a peer learning network. They were unaware that they had access to an existing peer learning network set up by NSW Department of Education on a Teams Channel	*“So, a network that you get together with a mix of schools who’ve done the initial training. Perhaps there’s a check in where the teachers can look at what they’ve implemented, what’s working, what’s not? Ask questions… I think that networking is really, really important.*” P17, Principal, regional school [in response to the researcher telling the participant there is a peer learning network on Teams], *“I didn’t know that one.”* P20, Teacher, regional school
Remind school personnel	Unfeasible	E-newsletters kept getting lost in principals’ inbox and were not being delivered to teachers, their target audience	*“They [the e-newsletters] do get lost. Well, lots of things get lost in my inbox because it’s ticking over all of the time.”* P17, Principal, regional school
Continuous progress monitoring	Not utilised	No participant reported utilising the self-report survey or fidelity self-checklist recommended for continuous progress monitoring	Analysis of the self-report survey data on Qualtrics online survey platform revealed that no participants completed the self-report survey

#### Refinements

The way in which staff feedback informed the reformatting of five underutilised strategies (Table 3) and the addition of a new strategy (all-in-one list of resources) for the second iteration of the PAX Plus implementation toolkit (version 2.0) is explained below.

**Table 3:** Comparison of strategies from original and refined toolkits highlighting changes made after acceptability testing

Original toolkit (v1)		Refined toolkit (v2)		
Strategy	Format	Retained/refined /added	Format	Quote justifying refinement
Audit and provide feedback	Recommend leadership team use aggregate school data, PAX Minutes or PAX scoreboard to monitor progress and provide feedback to teachers as a motivational tool	Retained	Recommend leadership team use aggregate school data, PAX Minutes or PAX scoreboard to monitor progress and provide feedback to teachers as a motivational tool	N/A
Executive support	Monthly meetings between researchers and school leadership team to report progress and problem solve implementation challenges	Retained	Monthly meetings between researchers and school leadership team to report progress and problem solve implementation challenges	N/A
School champions	Self-selecting staff member responsible for promoting program, coordinating activities, and distributing resources	Retained	Self-selecting staff member responsible for promoting program, coordinating activities, and distributing resources	N/A
Peer learning network	NSW Department of Education set up Microsoft Teams channel connecting staff from different schools trained in PAX GBG	Refined	Same channel promoted more explicitly using desk calendar and all-in-one list of resources	*“They [the teachers] don’t have the time to sit and go through the resources. They’d be missing a whole lot of resources that could be amazing, but it’s still the time to sit and go through and find them*.” P16, Principal
Recognition system	Recommend principal reward staff for their implementation efforts through a note on the tootle board or certificate of achievement	Refined	Recommend principal reward staff for their implementation efforts through a note on the tootle board, certificate of achievement or PAX branded t-shirt	*“I want a shirt…because I know you want to reward teachers. You could have a reward program where they [the teachers] could be nominated [for good program implementation] and they could get a shirt.”* P18, Teacher *“Well, that'd [the t-shirt] be fun as an incentive for the [program implementation].”* P28, Teacher
Remind school personnel	Monthly e-newsletters containing practical tips focused on specific program elements	Refined	Desk calendar containing practical tips focused on specific program elements	*“Yeah, that sounds good [the desk calendar] …because if it's there it's just that, "If I did that, that'd work," or, "That idea's good, but I would do…"* P14, Relief Teacher
Continuous progress monitoring	Fidelity self-checklist and/or online self-report survey to monitor progress	Refined	Walk-through observations of teachers’ program implementation	*“So, teachers have two observations per year… we as the observer, go in, have a look at the lesson, then we have a conversation with the teacher afterwards about “These are the things that we saw that worked really well”* P13, Principal
Promoting PAX Chats	Toolkit encourages principals to remind staff to participate in PAX Chats	Refined	NSW Department of Education and researchers remind staff to participate in refresher training integrated into PAX Chats	“*It was just a lot of information in a short period of time…Maybe something like little review videos would help*.” P4, Teacher “*I think that would be helpful to revitalize it (training) in the Australian format…that would be good as a refresher… Because the ones (demonstrative videos) that they have online are all American. If we could have some Australian ones”,* P29, Teaching Principal
All-in-one resources	N/A	Added	Email principals one page document listing available support resources. Encourage them to forward to staff	*“I think time is my biggest enemy…So if you can put things onto a one page with all of the links, I think that would be probably the most useful thing, because then they can save that page and go back to it when they want to either follow the link to a resource.”* P16, Principal

#### Recognition System

To improve the acceptability of the recognition system, one interviewee suggested using PAX-branded t-shirts to reward teachers. Other teachers agreed that PAX branded t-shirts would be a fun incentive to motivate adoption. The refined toolkit (version 2.0) encouraged principals to select the recognition system most appropriate for their school, with the option of using PAX-branded t-shirts in addition to ‘tootle boards for teachers’ and/or ‘certificates of achievement’ from the original toolkit (version 1.0).

#### Remind School Personnel

Teachers and the research team decided to reformat the e-newsletter into a physical flip-through desk calendar to improve the acceptability of the reminder system. Teachers felt the calendar would be a helpful visual reminder to deliver PAX GBG daily.

#### Continuous Progress Monitoring

Program champions suggested reformatting the continuous progress monitoring system from using self-report methods to walk-through observations. This involves the champion observing the teacher’s PAX GBG program implementation, assessing fidelity against a checklist, and providing feedback for improvement after class. Participants recommended walk-through observations be conducted ‘twice a term for 15 min each’.

#### Peer Learning Network

Educational staff wanted to use a peer learning network and were mostly unaware that an existing network was available. To raise awareness, information about accessing the peer learning network was provided in the desk calendar and ‘all-in-one’ list of resources.

#### Promoting PAX Chats/Refresher Training

PAX Chats were reformatted into short refresher training sessions. Educational staff asked for refresher videos to revise lessons from the initial workshop, which was considered too much information to digest in one day. Teachers wanted these videos to demonstrate program delivery in an Australian context. Leadership staff suggested it would be efficient to integrate refresher training into pre-recorded PAX Chats and share these with teachers during monthly staff meetings.

#### All-in-one List of Resources

Teachers wanted streamlined resources to support PAX GBG implementation. Specifically, several teachers wanted to receive a one-page email with a list of hyperlinked resources to save them the time of trawling through websites to find support materials.

#### Final PAX Plus Implementation Toolkit

The version 2.0 of PAX Plus implementation toolkit was distributed to principals of intervention schools on the 28th of February 2022. School recruitment and baseline data collection was completed in April 2022. The leadership team were responsible for selecting the most relevant strategies for supporting PAX GBG in their school and then sharing the resources from the toolkit to teachers and support staff.

Of the nine strategies offered in the final toolkit (Table 3), six targeted teachers’ motivation, and self-efficacy with the PAX GBG. These included desk calendar reminders, promotion of the peer learning network, PAX-branded t-shirts for recognition, PAX Chats/refresher training, audit and provide feedback, and an all-in-one list of resources. Three school-level strategies were provided to support school leaders to resolve implementation challenges (through monthly meetings), use program champions, and monitor progress using walkthrough observations.

## Discussion

Across three stages, a multicomponent toolkit (PAX Plus) was co-designed with educational staff, researchers, and Department of Education wellbeing staff to enhance the implementation of the PAX GBG program in NSW government primary schools. The PAX Plus implementation toolkit was grounded in implementation theory and scientific evidence to enhance potential effectiveness. At the completion of the iterative co-design process, PAX Plus was found to be acceptable to educational staff.

We identified several benefits of co-design, found in other trials of school-based programs. Involving educational staff from conception aligned PAX Plus to the needs of schools (Ponsford et al., 2021). Educational staff provided unique insights into the delivery format and timing of strategies that had worked in practice. Collaborating with the Department of Education wellbeing leadership team during the preparation phase also highlighted which evidence-based strategies were likely to be infeasible because of time and human resource considerations. This minimised research and effort waste by ensuring resources were not allocated to the development and evaluation of strategies that would not be feasible and thus ineffective in practice (Milton et al., 2022).

We found a slight disconnect between some of the strategies educators selected during the preparation phase and the strategies they used and preferred during implementation. During preparation, educational staff consistently asked for a peer learning network and access to PAX Chats, however after six months of implementation, few had utilised these strategies. From the interviews, it was clear that staff lacked awareness of the peer learning network and PAX Chats and felt they had no time to utilise the strategies. The outcomes of the co-design phase could have been improved by focusing not only on what strategies educational staff wanted, but how they wanted to be informed and reminded of these strategies. Our findings also demonstrate that strategy acceptability is dynamic, changing from preparation to implementation phases (Proctor et al., 2009). For example, whilst staff found a continuous monitoring system acceptable during preparation, it is possible that time constraints made this strategy unacceptable and underutilised during implementation. Our findings also suggest that acceptability alone does not predict strategy utilisation, rather there are other important factors such as appropriateness and feasibility (Weiner et al., 2017).

Executive support was found to be acceptable to educational staff, which involved the researcher acting as a consultant and providing monthly check-ins to the leadership team. There was some overlap between our executive support strategy and the coaching/consultation strategy found to improve implementation of the PAX GBG and other school-based mental health programs (Becker et al., 2013; Schneider et al., 2016; Stormont et al., 2015). It is best practice for consultants to be school psychologists, behavioural consultants, or other educational staff as they have lived experience and relatability to support program implementation (Erchul & Sheridan, 2014; Schneider et al., 2016; Streimann et al., 2017). However, during implementation trials like ours, it is much more common and feasible for consultation to be delivered by researchers (Stormont et al., 2015). We need to consider how monthly check-ins can be transferred to educational staff for program sustainability, without adding to the administrative burden facing Australian educators (Thomson & Hillman, 2019).

Program champions and monitor and provide feedback strategies were highly acceptable to educators, consistent with other school-based trials (Dijkman et al., 2017; Nadeem et al., 2018). Educational staff wanted school champions to be self-selecting program advocates, as seen elsewhere (Nadeem et al., 2018). Educational staff found it motivating to receive feedback about students’ behavioural outcomes, consistent with evidence that providing feedback has a moderate to large effect on program fidelity (Merle et al., 2022; Solomon et al., 2012). Our insight into the positive impact of monitoring and feedback on acceptability extends prior descriptions of this strategy to support the PAX GBG in Estonia (Streimann et al., 2020) and Denmark (Breeman et al., 2016).

We found Australian teachers wanted refresher training to reinforce the skills and knowledge acquired during initial training, as seen in the UK (Coombes et al., 2016), and for this training to be culturally adapted to include localised examples of implementation. Similar locally relevant adaptations have been used in Estonia (Streimann et al., 2017) and Canada (Wu et al., 2019) to improve the relevance of the US-developed training.

### Strengths and Limitations

A core strength of PAX Plus is that it was co-designed from conception with a range of senior and classroom-level educators and wellbeing staff. Taking a co-designed approach improves the likelihood of PAX Plus being effective (Milton et al., 2022), resulting in better outcomes for children and adolescents (Proctor et al., 2009).

Our multimethod qualitative approach was a strength. Focus group discussions allowed participants to build upon each other’s ideas of strategies that could improve PAX GBG program adoption (Stewart et al., 2007). This had a ‘synergetic effect’, resulting in co-design ideas that might not have been produced through one-on-one interviews (Stewart et al., 2007). During acceptability testing, it was appropriate to use semi-structured interviews to gain in-depth insights into how and why PAX Plus could be improved. In reporting, our findings were justified with data, a strength that distinguished our study from other co-design studies (Bruns et al., 2016; Kern et al., 2015). Lastly, our reporting of the co-design process was relatively in-depth compared to other studies, improving transparency and replicability (Becker et al., 2013; Coles et al., 2015).

The study had several limitations. First, it would have been beneficial to involve educators in phase 1 of the co-design, rather than only working with the Department of Education. Greater representation of stakeholders from all levels of the educational hierarchy would have allowed for better power distribution among those expected to do the work and those with strategic responsibility for the work. Second, our co-design process disproportionately involved regional schools, as regional schools were less affected by COVID-19 restrictions (e.g., school closures) during data collection, compared to urban schools. Certain strategies could be better suited to the characteristics of regional schools, such as truncating the role of principal and champions to accommodate for smaller school sizes. Our upcoming realist evaluation will consider what strategies worked to support PAX GBG implementation in different school settings and why.

## Conclusion

We co-designed a novel multicomponent implementation toolkit (PAX Plus) with educational staff and Department of Education leaders that shows promise for enhancing the implementation of school-based mental health prevention programs. The effects of PAX Plus on implementation and effectiveness outcomes are currently being tested using a cluster randomised hybrid effectiveness-implementation trial. This study demonstrates how involving educational stakeholders in co-design improves the acceptability and feasibility of implementation strategies, although acceptability is not always sufficient for adoption. The clear description of our co-design process provides a roadmap for other researchers and practitioners to co-design strategies with educational staff to enhance school-based program implementation.

### Supplementary Information

Below is the link to the electronic supplementary material.

Supplementary file1 (PDF 228 kb)

Supplementary file2 (PDF 243 kb)

Supplementary file3 (PDF 550 kb)

Supplementary file4 (DOC 21 kb)

## Abbreviations

NSW: New South Wales
PAX GBG: PAX Good Behaviour Game

## References

[CR1] Allara E, Beccaria F, Molinar R, Marinaro L, Ermacora A, Coppo A, Faggiano F (2019). A school-based program to promote well-being in preadolescents: Results from a cluster quasi-experimental controlled study. The Journal of Primary Prevention.

[CR2] Australian Curriculum, Assessment and Reporting Authority (ACARA). (2018). *Personal and social capability learning continuum*. https://www.australiancurriculum.edu.au/media/1078/general-capabilities-personal-and-social-capability-learning-continuum.pdf

[CR3] Baffsky R, Ivers R, Cullen P, Batterham PJ, Toumbourou J, Calear A, Werner-Seidler A, McGillivray L, Torok M (2022). A cluster randomised effectiveness-implementation trial of an intervention to increase the adoption of PAX Good Behaviour Game, a mental health prevention program, in Australian primary schools: Study protocol. Contemporary Clinical Trials Communications.

[CR4] Baffsky R, Ivers RQ, Cullen P, Wang J, McGillivray L, Torok M (2022). Strategies for enhancing the implementation of universal mental health prevention programs in schools: A systematic review. Prevention Science.

[CR5] Barnes C, McCrabb S, Stacey F, Nathan N, Yoong SL, Grady A, Sutherland R, Hodder R, Innes-Hughes C, Davies M (2021). Improving implementation of school-based healthy eating and physical activity policies, practices, and programs: A systematic review. Translational Behavioral Medicine.

[CR6] Becker KD, Bradshaw CP, Domitrovich C, Ialongo NS (2013). Coaching teachers to improve implementation of the good behavior game. Administration Policy in Mental Health and Mental Health Services Research.

[CR7] Bowles T, Jimerson S, Haddock A, Nolan J, Jablonski S, Czub M, Coelho V (2017). A review of the provision of social and emotional learning in Australia, the United States, Poland, and Portugal. Journal of Relationships Research.

[CR8] Breeman LD, van Lier PA, Wubbels T, Verhulst FC, van der Ende J, Maras A, Struiksma AC, Hopman JA, Tick NT (2016). Effects of the Good Behavior Game on the behavioral, emotional, and social problems of children with psychiatric disorders in special education settings. Journal of Positive Behavior Interventions.

[CR9] Bruhn AL, Hirsch SE, Lloyd JW (2015). Treatment integrity in school-wide programs: A review of the literature (1993–2012). The Journal of Primary Prevention.

[CR10] Bruns EJ, Duong MT, Lyon AR, Pullmann MD, Cook CR, Cheney D, McCauley E (2016). Fostering SMART partnerships to develop an effective continuum of behavioral health services and supports in schools. American Journal of Orthopsychiatry.

[CR11] Coelho VA, Sousa V, Figueira AP (2016). The effectiveness of a portuguese elementary school social and emotional learning program. The Journal of Primary Prevention.

[CR12] Coles EK, Owens JS, Serrano VJ, Slavec J, Evans SW (2015). From consultation to student outcomes: The role of teacher knowledge, skills, and beliefs in increasing integrity in classroom management strategies. School Mental Health.

[CR13] Collie RJ, Martin AJ, Frydenberg E, Frydenberg E, Martin AJ, Collie RJ (2017). Social and emotional learning: A brief overview and issues relevant to Australia and the Asia-Pacific. Social and emotional learning in Australia and the Asia Pacific.

[CR14] Coombes L, Chan G, Allen D, Foxcroft DR (2016). Mixed-methods evaluation of the good behaviour game in English primary schools. Journal of Community & Applied Social Psychology.

[CR15] Curran GM, Bauer M, Mittman B, Pyne JM, Stetler C (2012). Effectiveness-implementation hybrid designs: Combining elements of clinical effectiveness and implementation research to enhance public health impact. Medical Care.

[CR16] Dijkman MA, Harting J, van Tol L, Van der Wal MF (2017). Sustainability of the good behaviour game in Dutch primary schools. Health Promotion International.

[CR17] DuPaul GJ, Kern L, Belk G, Custer B, Hatfield A, Daffner M, Peek D (2018). Promoting parent engagement in behavioral intervention for young children with ADHD: Iterative treatment development. Topics in Early Childhood Special Education.

[CR18] Erchul WP, Sheridan SM, Erchul WP, Sheridan SM (2014). Overview: The state of scientific research in school consultation. Handbook of Research in School Consultation.

[CR19] Gale NK, Heath G, Cameron E, Rashid S, Redwood S (2013). Using the framework method for the analysis of qualitative data in multi-disciplinary health research. BMC Medical Research Methodology.

[CR20] Gilbert R (2019). General capabilities in the Australian curriculum: Promise, problems and prospects. Curriculum Perspectives.

[CR21] Hawkins J, Madden K, Fletcher A, Midgley L, Grant A, Cox G, Moore L, Campbell R, Murphy S, Bonell C (2017). Development of a framework for the co-production and prototyping of public health interventions. BMC Public Health.

[CR22] Herlitz L, MacIntyre H, Osborn T, Bonell C (2020). The sustainability of public health interventions in schools: a systematic review. Implementation Science.

[CR23] Kern L, Evans SW, Lewis TJ, State TM, Weist MD, Wills HP (2015). CARS comprehensive intervention for secondary students with emotional and behavioral problems: Conceptualization and development. Journal of Emotional and Behavioral Disorders.

[CR24] Kessler RC, Amminger GP, Aguilar-Gaxiola S, Alonso J, Lee S, Ustun TB (2007). Age of onset of mental disorders: A review of recent literature. Current Opinion in Psychiatry.

[CR25] Laurens KR, Graham LJ, Dix KL, Harris F, Tzoumakis S, Williams KE, Schofield JM, Prendergast T, Waddy N, Taiwo M (2021). School-based mental health promotion and early intervention programs in New South Wales, Australia: Mapping practice to policy and evidence. School Mental Health.

[CR26] Locke J, Lee K, Cook CR, Frederick L, Vázquez-Colón C, Ehrhart MG, Aarons GA, Davis C, Lyon AR (2019). Understanding the organizational implementation context of schools: A qualitative study of school district administrators, principals, and teachers. School Mental Health.

[CR27] Lyon AR, Bruns EJ (2019). From evidence to impact: Joining our best school mental health practices with our best implementation strategies. School Mental Health.

[CR28] Merle JL, Thayer AJ, Larson MF, Pauling S, Cook CR, Rios JA, McGinnis JL, Sullivan MM (2022). Investigating strategies to increase general education teachers' adherence to evidence-based social-emotional behavior practices: A meta-analysis of the single-case literature. Journal of School Psychology.

[CR29] Milton K, Poole K, Cross A, Gasson S, Gokal K, Lyons K, Pulsford R, Jones A (2022). ‘People don't get cancer, families do’: Co-development of a social physical activity intervention for people recently affected by a cancer diagnosis. European Journal of Cancer Care..

[CR30] Nadeem E, Saldana L, Chapman J, Schaper H (2018). A mixed methods study of the stages of implementation for an evidence-based trauma intervention in schools. Behavior Therapy.

[CR31] Nathan N, Yoong SL, Sutherland R, Reilly K, Delaney T, Janssen L, Robertson K, Reynolds R, Chai LK, Lecathelinais C (2016). Effectiveness of a multicomponent intervention to enhance implementation of a healthy canteen policy in Australian primary schools: a randomised controlled trial. International Journal of Behavioral Nutrition and Physical Activity.

[CR32] Owens JS, Lyon AR, Brandt NE, Warner CM, Nadeem E, Spiel C, Wagner M (2014). Implementation science in school mental health: Key constructs in a developing research agenda. School Mental Health.

[CR33] Pérez V, Rodríguez J, De la Barra F, Fernández AM (2005). Efectividad de una estrategia conductual para el manejo de la agresividad en escolares de enseñanza básica. Psykhe.

[CR34] Ponsford R, Meiksin R, Bragg S, Crichton J, Emmerson L, Tancred T, Tilouche N, Morgan G, Gee P, Young H (2021). Co-production of two whole-school sexual health interventions for English secondary schools: Positive choices and project respect. Pilot and Feasibility Studies.

[CR35] Proctor EK, Landsverk J, Aarons G, Chambers D, Glisson C, Mittman B (2009). Implementation research in mental health services: An emerging science with conceptual, methodological, and training challenges. Administration and Policy in Mental Health and Mental Health Services Research.

[CR36] Proctor EK, Silmere H, Raghavan R, Hovmand P, Aarons G, Bunger A, Griffey R, Hensley M (2011). Outcomes for implementation research: Conceptual distinctions, measurement challenges, and research agenda. Administration and Policy in Mental Health and Mental Health Services Research.

[CR37] Productivity Commission. (2020). *Mental health, inquiry report*. https://www.pc.gov.au/inquiries/completed/mental-health/report

[CR38] Saigh PA, Umar AM (1983). The effects of a good behavior game on the disruptive behavior of Sudanese elementary school students. Journal of Applied Behavior Analysis.

[CR39] Schneider DR, Pereira APD, Cruz JI, Strelow M, Chan G, Kurki A, Sanchez ZM (2016). Evaluation of the implementation of a preventive program for children in Brazilian schools. Psicologia Ciência e Profissão.

[CR40] Simpson JN, Hopkins S, Eakle CD, Rose CA (2020). Implement today! Behavior management strategies to increase engagement and reduce challenging behaviors in the classroom. Beyond Behavior.

[CR41] Smith EP, Osgood DW, Oh Y, Caldwell LC (2018). Promoting afterschool quality and positive youth development: Cluster randomized trial of the PAX Good Behavior Game. Prevention Science.

[CR42] Solomon BG, Klein SA, Politylo BC (2012). The effect of performance feedback on teachers' treatment integrity: A meta-analysis of the single-case literature. School Psychology Review.

[CR43] Stewart DW, Shamdasani PN, Rook DW, Stewart DW, Shamdasani PN, Rook DW (2007). Group dynamics and focus group research. Focus Groups: Theory and Practice.

[CR44] Stokols D (2006). Toward a science of transdisciplinary action research. American Journal of Community Psychology.

[CR45] Stormont M, Reinke WM, Newcomer L, Marchese D, Lewis C (2015). Coaching teachers’ use of social behavior interventions to improve children’s outcomes: A review of the literature. Journal of Positive Behavior Interventions.

[CR46] Streimann K, Selart A, Trummal A (2020). Effectiveness of a universal, classroom-based preventive intervention (PAX GBG) in Estonia: A cluster-randomized controlled trial. Prevention Science.

[CR47] Streimann K, Trummal A, Klandorf K, Akkermann K, Sisask M, Toros K, Selart A (2017). Effectiveness of a universal classroom-based preventive intervention (PAX GBG): A research protocol for a matched-pair cluster-randomized controlled trial. Contemporary Clinical Trials Communications.

[CR48] Sutherland R, Campbell E, McLaughlin M, Nathan N, Wolfenden L, Lubans DR, Morgan PJ, Gillham K, Oldmeadow C, Searles A (2020). Scale-up of the physical activity 4 everyone (PA4E1) intervention in secondary schools: 12-month implementation outcomes from a cluster randomized controlled trial. International Journal of Behavioral Nutrition and Physical Activity.

[CR49] Thomson, S., & Hillman, K. (2019). *The teaching and learning international survey 2018. Australian report volume 1: Teachers and school leaders as lifelong learners*. https://research.acer.edu.au/cgi/viewcontent.cgi?article=1007&context=talis

[CR50] Weiner BJ, Lewis CC, Stanick C, Powell BJ, Dorsey CN, Clary AS, Boynton MH, Halko H (2017). Psychometric assessment of three newly developed implementation outcome measures. Implementation Science.

[CR51] Werner-Seidler A, Spanos S, Calear AL, Perry Y, Torok M, O'Dea B, Christensen H, Newby JM (2021). School-based depression and anxiety prevention programs: An updated systematic review and meta-analysis. Clinical Psychology Review.

[CR52] Williams I, Vaisey A, Patton G, Sanci L (2020). The effectiveness, feasibility and scalability of the school platform in adolescent mental healthcare. Current Opinion in Psychiatry.

[CR53] Wolfenden L, Nathan N, Reilly K, Delaney T, Janssen LM, Reynolds R, Sutherland R, Hollis J, Lecathelinais C, Williams C (2019). Two-year follow-up of a randomised controlled trial to assess the sustainability of a school intervention to improve the implementation of a school-based nutrition policy. Health Promotion Journal of Australia.

[CR54] Wu YQ, Chartier M, Ly G, Phanlouvong A, Thomas S, Weenusk J, Murdock N, Munro G, Sareen J (2019). Qualitative case study investigating PAX-good behaviour game in first nations communities: Insight into school personnel’s perspectives in implementing a whole school approach to promote youth mental health. British Medical Journal Open.

