# Supplementary material for: Co-design and Development of Implementation Strategies: Enhancing the PAX Good Behaviour Game in Australian Schools
Source: J Prev (2022). 2023 Sep 23;44(6):679–704. doi: 10.1007/s10935-023-00749-9 (PMC10638156; doi:10.1007/s10935-023-00749-9)
Supplement: Supplementary file 1 — Supplementary file1 (PDF 228 kb) [file 10935_2023_749_MOESM1_ESM.pdf]

Supplementary File 1: Educational staff involved in stages 2 and 3 of co-design

The 15 government primary schools involved in stages 2 and 3 of the co-design process were from Greater Western Sydney, Central Coast, Riverina, Northern Tablelands, Central Tablelands, Southern Tablelands, Coffs Harbour, Greater Newcastle and Hunter regions of New South Wales, Australia. Schools were within the 2nd to 73rd percentile of socio-educational advantage compared to other NSW schools according to the School Index of Community Socio-Educational Advantage. We worked with a total of 29 educational staff (see table 1), including 13 classroom teachers, 7 school principals, 8 assistant principals/teachers and 1 instructional leader. Ninety-three percent of staff were female.

Table 1

Role of educational staff (N=29) involved in the co-design

Educational staff	Stage 2. Co-design	Stage 3. Acceptability testing	Staff who took part in both stages	Total
Role				
Classroom Teacher	7	6	0	13
Principal	3	6	2	7
Assistant Principal/teacher	6	3	1	8
Instructional Leader	0	1	0	1
Total	16	16	3	29
