# Supplementary material for: Co-design and Development of Implementation Strategies: Enhancing the PAX Good Behaviour Game in Australian Schools
Source: J Prev (2022). 2023 Sep 23;44(6):679–704. doi: 10.1007/s10935-023-00749-9 (PMC10638156; doi:10.1007/s10935-023-00749-9)
Supplement: Supplementary file 2 — Supplementary file2 (PDF 243 kb) [file 10935_2023_749_MOESM2_ESM.pdf]

Supplementary File 2: Rapid review search strategy

PubMed, PsycINFO, CINAHL and ERIC

**Concept**	**Key words**
Mental Health	"mental health" OR "mental healthcare" OR "public mental health" OR "behavior problems" OR "externalizing symptoms" OR "internalizing symptoms" OR "mental health" OR "behavioural problems" OR "behaviour problems" OR "social-emotional" OR "Social Emotional Learning" OR "behavioral difficulties" OR "behavioral problems"
School	School* OR student* or teacher* or classroom* or pupil* "primary school" OR "primary education" OR "elementary school" OR school-based"
Intervention	intervention* OR "preventative mental health service" or program* or "evidence based practice" OR EBP*
Implementation	implementation OR "Implementation Science" OR uptake OR sustainability OR feasibility OR reach OR appropriateness OR barriers OR facilitators OR fidelity OR adoption OR acceptability
NOT	NOT college OR "high school" OR "secondary school" OR "tertiary education" OR "higher education"
NOT	"hospital" OR "primary health care" OR "primary care" OR hospital-based OR "general practitioner" OR nurse OR "family medicine" OR "general medicine" OR nurse OR clinician

Limit results to 2000-2021, English and Journal articles.

Google Scholar:

"mental health" "prevention program" "implementation strategy" children ("elementary school" OR "primary school"

Limit results to 2000-2021
