# Supplementary material for: Co-design and Development of Implementation Strategies: Enhancing the PAX Good Behaviour Game in Australian Schools
Source: J Prev (2022). 2023 Sep 23;44(6):679–704. doi: 10.1007/s10935-023-00749-9 (PMC10638156; doi:10.1007/s10935-023-00749-9)
Supplement: Supplementary file 3 — Supplementary file3 (PDF 550 kb) [file 10935_2023_749_MOESM3_ESM.pdf]

Supplementary File 3. Rapid review PRISMA flow diagram

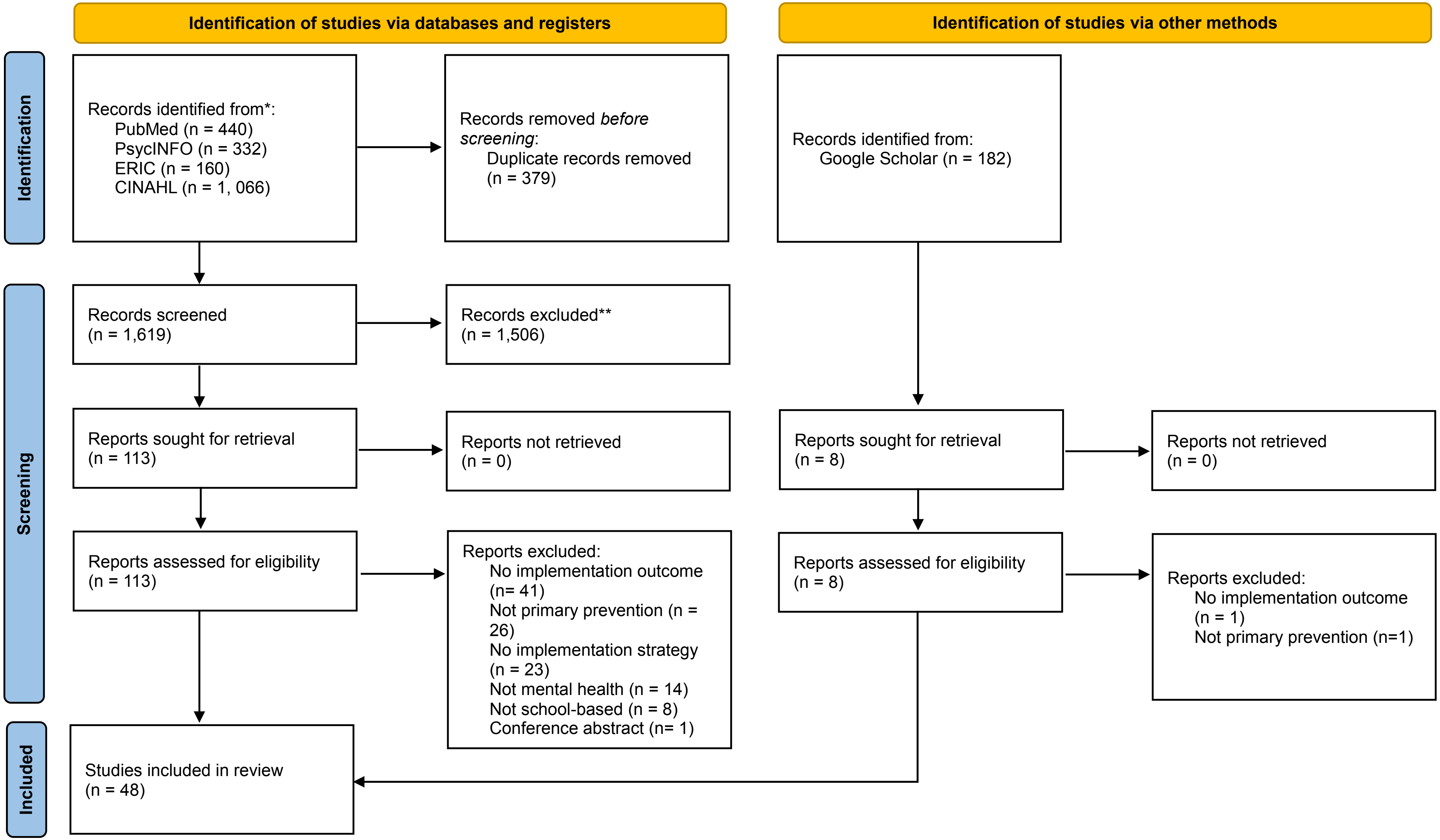

From: Page MJ, McKenzie JE, Bossuyt PM, Boutron I, Hoffmann TC, Mulrow CD, et al. The PRISMA 2020 statement: an updated guideline for reporting systematic reviews. BMJ 2021;372:n71. doi: 10.1136/bmj.n71. For more information, visit: http://www.prisma-statement.org/
