# Supplementary material for: Co-design and Development of Implementation Strategies: Enhancing the PAX Good Behaviour Game in Australian Schools
Source: J Prev (2022). 2023 Sep 23;44(6):679–704. doi: 10.1007/s10935-023-00749-9 (PMC10638156; doi:10.1007/s10935-023-00749-9)
Supplement: Supplementary file 4 — Supplementary file4 (DOC 21 kb) [file 10935_2023_749_MOESM4_ESM.docx]

**Supplementary file 4: Draft strategies identified in rapid review and discussed with NSW Department of Education**

********strategies NSW Department of Education leadership team found useful*

|  | ***Strategy*** | **What this is?** | **Evidence** | **Resource burden to implement** |
| --- | --- | --- | --- | --- |
| *1* | ***School champions**** | Having one or more staff members who advocate for the program within the school. | A number of studies have consistently shown this strategy increases program adoption and sustainability when combined with other strategies (such as educational materials) (Nathan et al., 2016; Mathur et al., 2016; Lytle et al., 2006; De Villiers et al., 2015; Dijkman et al., 2017; Nadeem et al., 2018). | Low resource burden. Involves training one person per school and this would not require additional funding. |
| 2 | ***Audit and provide feedback**** | Providing teachers with feedback on how they are doing. For example, informing them how their program delivery is impacting student outcomes. | A number of studies show that consultants providing feedback increases teachers’ program adoption, fidelity and sustainability which in turn leads to greater improvements in children’s outcomes (Farmer-Dougan et al., 1999; Jones et al., 1997; Mortenson & Witt, 1998; Noell et al., 1997; Witt et al., 1997; Solomon et al., 2012; Wolfenden et al., 2017; Fallon et al., 2018; Hailemariam et al., 2019). | High resource burden. It would involve school-level de-identified analysis of implementation outcomes at 6 weeks, 6 months and 12 months. However, given strength of the evidence-base it could be a worthwhile effort. |
| *3* | ***Recognition system**** | Providing positive reinforcement to reward teachers for their implementation efforts. | One study shows that this strategy combined with others increased the sustainability of a school-based health eating intervention (Wolfenden et al., 2019). | Low resource burden. |
| *4* | ***Executive support**** | The research team communicate the importance and benefits of the program to principals who then endorse and promote it to teachers, parents and students. | Five studies found that principal support increased teachers’ implementation of school-based programs (Han & Weiss, 1995; Fullan et al., 1980; Gottfredson et al., 1997;  Gottfredson & Gottfredson, 2002; Rohrbach et al., 1993). | Low resource burden, however need to be mindful of not overburdening school leadership team. |
| *5* | ***Remind school personnel**** | Teachers are emailed a reminder about the PAX steps and asked to review the email reminder before implementing the game each day. Also provided with a ‘quick tip’ on how to improve delivery of one area. | Evidence from 3 studies in a systematic review that emailed reminders had a small effect on fidelity (Forman-Hoffman et al., 2017). | Low resource burden. |
| *6* | ***Implementation planning**** | Teacher and researchers do action/coping planning where they go through the logistics of each step of implementation and problem solve how to overcome potential barriers. | Seven studies have shown this strategy increases fidelity (implementing the program as intended) (Byron, Sanetti, & Kratochwill, 2018; Sanetti et al., 2014; Sanetti & Collier-Meek, 2015; Sanetti, Collier-  Meek, Long, Byron, & Kratochwill, 2015; Sanetti, Williamson, Long, & Kratochwill, 2018, Collier-Meek 2019). | High resource burden. Requires a lot of personnel time and given time constraints we could only offer implementation planning to a small number of teachers. |
| *7* | ***Motivational Interviewing*** | A counselling technique that focuses on strengthening the teachers’ motivation to implement a new program or commitment to change. | Inconsistent evidence regarding the effect of motivational interviewing on outcomes. Some evidence from Collier-Meek and colleagues (2019) that motivational interviewing leads to better fidelity. However, no evidence from Lyon and colleagues (2019) or Larson and colleagues. (2020) that it leads to better outcomes. | High resource burden. Requires a lot of personnel and funding to implement. |
| *8* | ***Marketing strategies*** | Teachers receive regular communication of key messages of the program. | Two studies show that this strategy combined with others increased the sustainability of a school-based physical health program (Wolfenden et al., 2019; McHugh & Barlow, 2010). Another study found teachers receiving social marketing strategies reported more confidence implementing a school-based physical health program compared to those in the standard delivery condition (Delk et al., 2014). | Medium resource burden. Personnel need to develop content and coordinate production and additional funds are needed to produce marketing materials. |
| *9* | ***Educational outreach*** | A trained person meets with educational staff in their classroom to educate them about the new program with the intention of motivating adoption. | Consistent, medium quality evidence- a number of studies show this strategy increased program adoption and integration when combined with other strategies (Naylor et al., 2006; Sallis et al., 1997; Whatley Blum et al., 2007; Wolfenden et al., 2017; Delk et al., 2014). | High resource burden. We do not have the funding for on-site travel. Also we do not have the personnel time to do monthly visits for the number of participants we would need to obtain sufficient power for the study. |
| 10 | ***Local Consensus Process*** | A researcher holds meetings with teachers to reach consensus on the goals of the program, how best to implement it and develop plans for teachers to coordinate implementation tasks. | Inconsistent evidence. Some studies showed this strategy increases program adoption and integration when combined with other strategies (Wolfenden et al., 2017; Saraf et al., 2015; Perry et al., 2004; Naylor et al., 2006; Nathan et al., 2016; Mathur et al., 2016; Lytle et al., 2006; French et al., 2004; Delk et al., 2014). Other studies showed this strategy had no significant effect on program adoption when combined with other strategies (Alaimo et al., 2015; McCormick et al., 1995; Young et al., 2008) | High resource burden, involves a lot of administrative support to coordinate meetings with teachers then research team time to conduct focus group discussions to reach consensus. |
| 11 | ***Conduct Educational Meetings*** | Hold meetings with various key stakeholders (teachers, principals, Department of Education officials, parents, community members)  to teach them about the new program. | A number of studies showed that education meetings increased program adoption when used in combination with other strategies such as executive support, audit and provide feedback and educational materials (Wolfenden et al., 2017; Sutherland et al., 2017; Sallis et al., 1997; Naylor et al., 2006). | High resource burden to participants, partners and researchers. Given time and personnel constraints, it is not feasible for educational staff to take time off school to attend educational meetings. |
